# Effects of Tai Chi Cloud Hands on balance and resting-state functional connectivity after stroke: an fNIRS study

**DOI:** 10.3389/fneur.2026.1791157

**Published:** 2026-05-07

**Authors:** Jincheng Li, Zhenghao Dong, Mingxue Fan, Xiang Wang, Yingli Bi, Zhezhe Ma, Shiyan Wang, Jin Wang, Zunke Gong

**Affiliations:** 1 Xuzhou Rehabilitation Hospital Affiliated to Xuzhou Medical University, Xuzhou, China; 2 The Second Clinical College, Xuzhou Medical University, Xuzhou, China; 3 Xuzhou Rehabilitation Hospital, Xuzhou, China; 4 Department of Rehabilitation Medicine, Xuzhou Central Hospital, Xuzhou, China; 5 Pizhou Hospital Affiliated to Xuzhou Medical University, Xuzhou, China

**Keywords:** balance, dorsolateral prefrontal cortex, functional near-infrared spectroscopy (fNIRS), premotor cortex, rehabilitation, resting-state functional connectivity, stroke, Tai Chi Cloud Hands

## Abstract

**Background:**

Many people experience balance problems after a stroke. It remains unclear how a short Tai Chi Cloud Hands programme affects resting-state connectivity within the prefrontal–motor network.

**Methods:**

This study included 52 inpatients with post-stroke balance problems. Participants were randomly assigned to receive either standard rehabilitation alone or standard rehabilitation plus Tai Chi Cloud Hands. Both groups completed 30 min of rehabilitation per day, 5 days per week, for 2 weeks. Additionally, the experimental group completed 30 min of Cloud Hands training daily for 2 weeks. Outcome measures were collected before and after the intervention by assessors blinded to group allocation. The primary outcome was the Berg Balance Scale (BBS), and secondary outcomes included the Modified Barthel Index (MBI). Static balance was assessed using the PROKIN system, which measured centre-of-pressure (COP) path length and sway area. Resting-state brain connectivity was assessed using functional near-infrared spectroscopy (fNIRS) to monitor oxygenated haemoglobin. Group differences were analysed using ANCOVA, with false discovery rate (FDR) correction applied to 28 predefined brain region pairs.

**Results:**

The experimental group showed greater improvements in BBS (partial η^2^ = 0.420; adjusted difference = 3.215 points) and MBI (partial η^2^ = 0.363; adjusted difference = 6.056 points) than the control group. Notable reductions were also observed in COP path length (partial η^2^ = 0.295; adjusted difference = −115.816 mm) and sway area (partial η^2^ = 0.112; adjusted difference = −79.480 mm^2^). After FDR correction, significant group differences in connectivity included stronger DLPFC–PreM and PreM–M1 coupling, stronger interhemispheric M1–M1 connectivity, and reduced SMA–SMA coupling (all *q* ≤ 0.047).

**Conclusion:**

Adding a 2-week, high-frequency Tai Chi Cloud Hands programme to standard rehabilitation, delivered at a higher total therapy dose, was associated with greater improvements in balance, daily function, and static postural stability. These improvements were observed alongside regionally specific changes in prefrontal–motor connectivity, including enhanced DLPFC–PreM and PreM–M1 coupling and reduced interhemispheric SMA connectivity. These connectivity findings should be interpreted cautiously as associative rather than mechanistic.

## 1 Introduction

Balance dysfunction affects approximately 50–80% of individuals after stroke (1, 2). It often manifests as unsteady standing, abnormal gait, and impaired postural control, thereby increasing fall risk and limiting daily activities and participation (3). Postural control depends on multisensory integration of visual, proprioceptive, and vestibular inputs, as well as coordinated neural processing to maintain orientation and equilibrium (4). At the cortical level, posture and gait control recruit distributed networks involving prefrontal and sensorimotor regions, as well as premotor (PreM) and supplementary motor areas (SMA) that support anticipatory and reactive adjustments (5). Under more demanding conditions, executive control and attentional allocation become particularly important, and executive function has been shown to independently relate to balance and mobility performance after stroke (6). Accordingly, interactions between prefrontal and motor-related networks are increasingly considered relevant to balance recovery mechanisms.

In clinical practise, balance rehabilitation is typically delivered as task-specific, progressive training within comprehensive stroke rehabilitation programmes (7). Beyond conventional therapy, technology-assisted approaches are used in some settings, including force-platform feedback system (8), virtual reality-based training (9), robotic or electromechanical gait-training devices (10), and non-invasive brain stimulation techniques such as transcranial direct current stimulation (11). However, limited resources and the need for specialised equipment or space can restrict access and reduce the achievable training dose; observational data further indicate that the amount of movement practise delivered during routine inpatient rehabilitation can be low (12). Inpatient rehabilitation is often time limited; therefore, practical and scalable approaches that can be implemented with minimal equipment and sustained after discharge remain needed.

Tai Chi Cloud Hands is a core, relatively standardised Tai Chi movement that combines trunk rotation, coordinated eye–hand tracking, symmetrical circular arm movements, and rhythmic weight shifting, and it can be delivered with minimal equipment (13). Growing randomised evidence suggests that Tai Chi Yunshou (Cloud Hands) training programmes—typically delivered over several weeks—can improve balance and motor outcomes after stroke, although effect sizes vary across trials and protocols (14). However, much of the neuroimaging evidence in stroke rehabilitation has focused on task-evoked cortical responses rather than resting-state network organisation (15). Resting-state functional near-infrared spectroscopy (fNIRS) offers an accessible approach for characterising functional connectivity and network topology based on spontaneous low-frequency hemodynamic fluctuations (16), although reliability depends on acquisition and analytic parameters such as scanning duration (17). Graph-based resting-state fNIRS analyses have also been used to characterise whole-brain functional network organisation in humans (18).

In this randomised controlled trial, we added a 2-week Tai Chi Cloud Hands programme to conventional rehabilitation. The primary outcome was the Berg Balance Scale (BBS). Secondary outcomes included the Modified Barthel Index (MBI) and two centre-of-pressure (COP)-based indices of static postural stability during quiet stance, namely COP path length and COP sway area, which are commonly derived from posturographic assessment and are sensitive to stance conditions (19). We also examined resting-state region-of-interest (ROI)-to-ROI functional connectivity of oxygenated haemoglobin (HbO) within the prefrontal–motor network using fNIRS. The aim was to determine whether a short course of Cloud Hands training provides additional benefit and to explore potential network-level connectivity patterns associated with balance recovery during a brief inpatient stay.

## 2 Materials and methods

### 2.1 Ethics and registration

The study was approved by the Medical Ethics Committee of Xuzhou Rehabilitation Hospital (No. XK-LSW-2025-027) and was filed in the National Medical Research Registration and Record System (No. MR-32-25-088223). During the trial registration process, the study was initially submitted through the Chinese clinical trial registration pathway. However, it was subsequently considered to fall within the category of traditional medicine clinical trials, and registration through the International Traditional Medicine Clinical Trial Registry platform was advised. Therefore, WHO primary registry registration was not completed before enrolment of the first participant. At the time of manuscript revision, the study had been submitted to the International Traditional Medicine Clinical Trial Registry platform and was under review.

### 2.2 Participants

We enrolled 52 inpatients with post-stroke balance impairment at Xuzhou Rehabilitation Hospital in Jiangsu, China. A stroke was confirmed by CT or MRI. Baseline demographic and clinical characteristics are presented in Table 1.

**Table 1:** Comparison of baseline characteristics between the groups.

Group	*n*	Sex (male/female), n	Age, years	Time since stroke, days	Stroke type (ischaemic/haemorrhagic), n	Hemiplegic side (left/right), n
Experimental	22	15/7	61.820 ± 5.422	27.270 ± 12.627	18/4	14/8
Control	22	16/6	60.000 ± 6.514	25.270 ± 17.874	16/6	13/9
*χ*^2^/*t* value		0.109	0.943	0.520	0.518	0.096
*p* value		0.741	0.351	0.606	0.472	0.757

Values are mean ± SD or n. *n* = 22 per group. Between-group comparisons used independent-samples *t*-tests or *χ*^2^ tests.

### 2.3 Inclusion criteria

(1) Age 40–75 years; (2) first-ever stroke, 2 weeks to 6 months since onset, and stable vital signs; (3) Brunnstrom stage IV or V in the affected upper limb and standing balance level 1 or 2, allowing supervised standing training; (4) Modified Ashworth Scale score below 2 for the upper limb; (5) clear consciousness, no major language or comprehension problems, a Montreal Cognitive Assessment (MoCA) score of at least 18, and ability to follow instructions; and (6) written informed consent.

### 2.4 Exclusion criteria

(1) Critical condition, unstable vital signs, or progressive or secondary stroke; (2) significant impairments in consciousness, hearing, vision, attention, or intellectual function that precluded training or assessment; (3) severe neuromuscular, skeletal, or joint disorders affecting movement, or healing fractures; (4) severe diabetes, hypertension, or major diseases such as heart, liver, or kidney failure, blood disorders, or cancer; and (5) lower-limb deep vein thrombosis or other conditions rendering standing training unsafe.

### 2.5 Withdrawal and dropout criteria

(1) Serious adverse events that made it unsafe to continue; (2) worsening of the condition or development of serious complications during the trial; (3) completion of less than 70% of the Tai Chi Cloud Hands training; or (4) more than 20% of fNIRS channels classified as low quality.

### 2.6 Changes from the earlier registered protocol

In an earlier registry version, the Fugl-Meyer Assessment (FMA) and the Limits of Stability (LOS) test were listed as study outcomes. After the pilot phase and before the start of the formal study, both outcomes were removed from the formal study protocol. FMA was removed because the formal study was further focused on balance-related outcomes. LOS was removed because pilot testing showed difficulty in data collection and a high risk of incomplete data in this patient population. Therefore, neither FMA nor LOS was collected in the formal study.

### 2.7 Grouping and interventions

The study participants were randomly assigned to the experimental or control group using computer-generated random numbers with a 1:1 allocation ratio. The sequence was generated before enrolment by an independent researcher using SPSS version 27.0 (IBM Corp., Armonk, NY, USA; Chinese version). Random numbers were generated with the Mersenne Twister random number generator and a fixed seed number of 20250217. Block randomisation with a block size of 4 was used to maintain balance between groups during enrolment. The randomisation sequence was retained by the same independent researcher, who was not involved in participant recruitment, eligibility screening, baseline assessment, intervention delivery, outcome assessment, or data analysis. Group assignments were disclosed only after enrolment and completion of baseline assessments, according to the prespecified randomisation list. The intervention involved observable physical training; therefore, participants and therapists could not be blinded.

#### 2.7.1 Control group

The control group received conventional balance rehabilitation, including postural control, weight shifting, dynamic and passive balance training, balance strategies for the hip, knee, and ankle, core stability exercises, and training for muscle strength and joint range of motion. Training was conducted once daily for 30 min, 5 days per week, over 2 weeks.

#### 2.7.2 Experimental group

In addition to conventional rehabilitation, the experimental group practised Tai Chi Cloud Hands for 30 min each day, 7 days per week, for 2 weeks. The protocol used Chen-style Tai Chi Cloud Hands, emphasising the rhythm of open, close, turn, pass, and return movements and coordination of trunk rotation with weight shifting. Participants stood with their feet shoulder-width apart, bent their knees at least 30°, and rotated their trunks at least 30° to move their arms in symmetrical arcs in front of the chest. They followed trunk and palm movements with their gaze and breathed slowly and deeply into the abdomen whilst focusing on “qi sinking to the dantian.” Each movement cycle lasted at least 6 s. Rest intervals were adjusted based on perceived exertion. The therapists followed a standardised intervention manual, and participants received the same sequence of instructions and progression criteria. During the intervention, caregivers and patients trained together, with caregivers supervising and recording the patients’ training times. Over the 2-week intervention period, the control group received approximately 300 min of conventional rehabilitation (30 min/day, 5 days/week), whilst the experimental group received approximately 720 min of total therapy, including 300 min of conventional rehabilitation and 420 min of Tai Chi Cloud Hands training.

### 2.8 Outcome assessment

All participants were assessed twice, before the intervention and after the 2-week intervention. Assessments were performed by an experienced attending physician who was not involved in randomisation, intervention delivery, or data analysis and remained blinded to group allocation and treatment details throughout the study. Participants were instructed not to disclose their group assignment or training content during the assessments.

#### 2.8.1 BBS

We used the BBS to assess balance. The BBS consists of 14 items with a maximum score of 56, with higher scores indicating better balance.

#### 2.8.2 MBI

We used the MBI to assess activities of daily living. The MBI includes 10 items with a maximum score of 100, with higher scores indicating greater independence.

#### 2.8.3 Static postural stability

We measured static postural control using the PROKIN balance assessment and training system (Tecnobody, Italy). Participants stood barefoot with their arms at their sides, looked forward, and maintained a quiet stance with eyes open for 30 s. The test was repeated three times, and the average value was used. The system automatically calculated COP path length and COP sway area. Lower values indicated better static postural control.

### 2.9 Safety monitoring

We monitored safety throughout the intervention and assessment periods. The prespecified safety monitoring items included vital signs, intervention-related symptoms or injuries, and fNIRS-related discomfort or adverse events. Vital signs, including heart rate, blood pressure, and oxygen saturation, were checked before and after each intervention session and when clinically indicated during training or assessment. Intervention-related adverse events included dizziness, headache, nausea, fatigue, limb or joint pain, muscle strain, and falls. fNIRS-related adverse events included visual discomfort, eye fatigue, and seizure. Any adverse event was recorded throughout the study period. Training or assessment was discontinued if participants developed serious dizziness, severe headache, falls, marked musculoskeletal pain or injury, clinical deterioration, recurrent stroke, or seizure.

### 2.10 fNIRS data acquisition and processing

**Figure 1:**
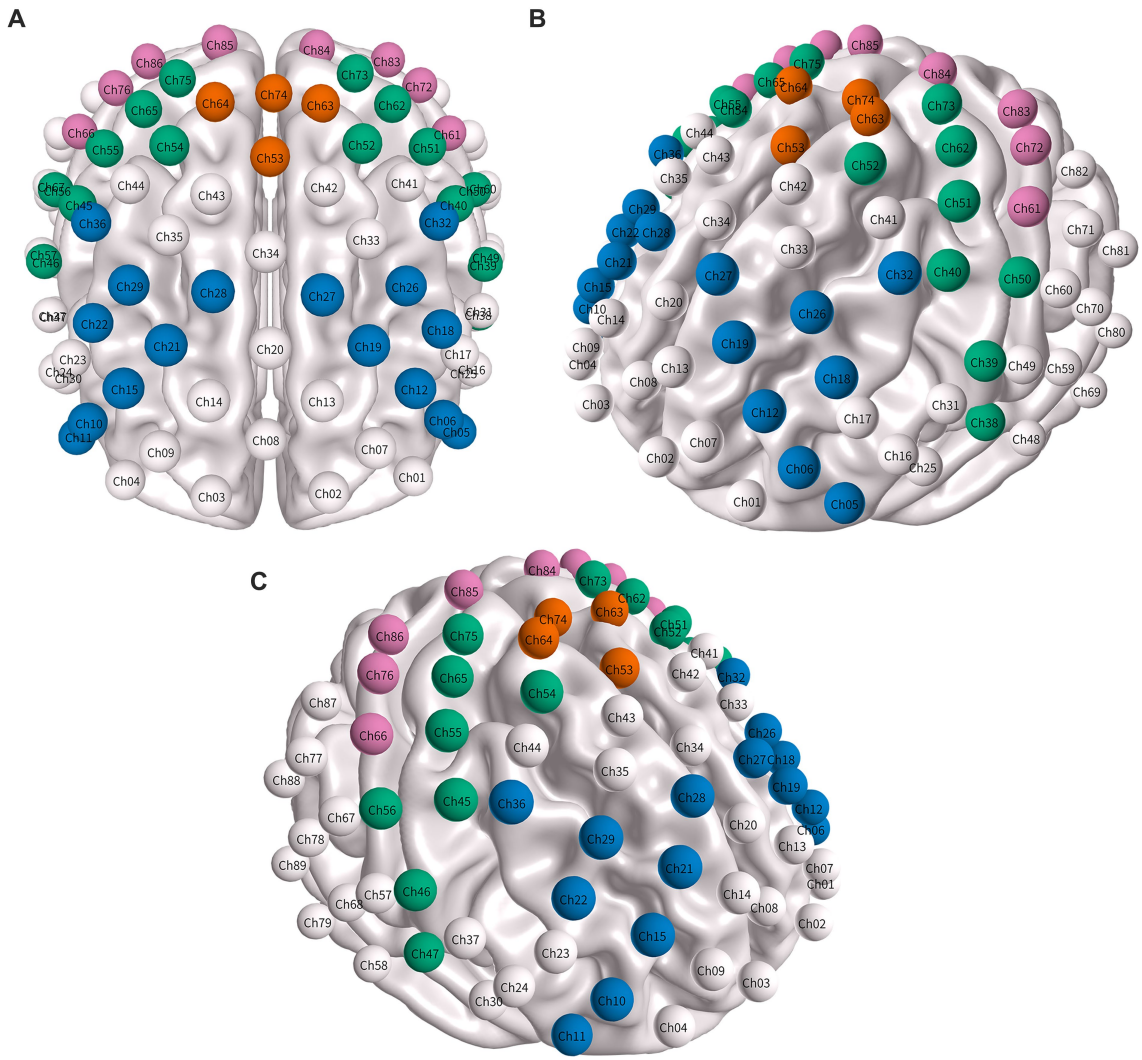
Three-dimensional mapping of regions of interest (ROIs). **(A)** Top (dorsal) view, **(B)** left lateral view, and **(C)** right lateral view of the 89-channel fNIRS montage with ROI locations overlaid. Channels belonging to each prespecified ROI are colour-coded for visualisation: DLPFC (blue), PreM (green), SMA (orange), and M1 (pink). Channel numbering and hemisphere assignment (left/right) follow the manufacturer’s montage. The full ROI–channel mapping is shown in Table 2.

We recorded resting-state fNIRS data using the BS-2000 fNIRS imaging system (Wuhan Zilian Hongkang Technology Co., Ltd.), which includes 24 sources and 32 detectors, yielding a total of 89 channels. Optodes were positioned according to the international 10–20 system, with approximately 30 mm between optodes. A 3D digitiser (NirMap, Wuhan Zilian Hongkang Technology Co., Ltd.) recorded reference points (Nz, Cz, AL, RL) and optode positions, which were subsequently mapped to a standard MNI cortical template. Eight ROIs were defined: bilateral dorsolateral prefrontal cortex (DLPFC), SMA, PreM, and M1 (see Figure 1 and Table 2). Continuous-wave signals were collected at wavelengths of 690 and 830 nm at a sampling rate of 10 Hz. Before data acquisition, participants rested quietly for 10 min. During recording, they kept their eyes closed, remained still, and attempted to avoid falling asleep. Approximately 6 min of resting-state data were collected (20). For each channel, the coefficient of variation (CV) was calculated, and channels with a CV greater than 15% were classified as low quality. Participants with more than 20% low-quality channels were excluded (21).

**Table 2:** Definition of regions of interest (ROIs) and channel composition.

ROI	Hemisphere	Channels
DLPFC	Left	5, 6, 12, 18, 19, 26, 27, 32
	Right	10, 11, 15, 21, 22, 28, 29, 36
PreM	Left	38, 39, 40, 50, 51, 52, 62, 73
	Right	45, 46, 47, 54, 55, 56, 65, 75
M1	Left	84, 61, 72, 83
	Right	86, 76, 66, 85
SMA	Left	63, 74
	Right	53, 64

Regions of interest (ROIs) were defined at the channel level based on the manufacturer’s 89-channel montage (YIRUIDE BS-2000, Wuhan, China). Hemisphere assignment (left/right) followed the vendor’s montage definition. This ROI–channel mapping was used consistently for all resting-state functional connectivity analyses. Schematic visualisations of ROI locations are shown in Figure 1.

Raw intensity data were processed using NirMaster (Wuhan Zilian Hongkang Technology Co., Ltd.). The preprocessing pipeline began by converting raw light intensity to optical density. Motion artefacts were corrected using spline interpolation to mitigate transient disturbances. A 0.01–0.1 Hz band-pass filter was applied to attenuate physiological noise and drift, followed by linear detrending. The modified Beer–Lambert law was used to calculate changes in HbO and deoxyhaemoglobin concentrations (22), with HbO selected as the primary indicator. The BS-2000 system used in this study did not include short-separation channels; therefore, short-channel regression was not performed. Global signal regression was also not applied. Within the NirMaster resting-state module, we extracted ROI-level HbO time series, calculated Pearson correlation coefficients for all ROI pairs, and applied Fisher r-to-z transformation. The resulting *Z* values represented functional connectivity strength. ROI-to-ROI connectivity matrices were also constructed for statistical analysis.

### 2.11 Sample size estimation

We estimated the sample size based on the expected group difference in BBS scores using baseline-adjusted ANCOVA. Post-intervention BBS served as the dependent variable, the group served as the fixed factor, and baseline BBS as the covariate. Clinical thresholds for the BBS (minimal detectable change of approximately 4–6 points and minimal important change of approximately 6 points) supported an expected between-group difference of 4 points (23). We assumed a BBS standard deviation of 6 and a baseline-to-post correlation of 0.70. Under ANCOVA, residual variance is reduced by a factor of (1 − ρ^2^), yielding an estimated residual standard deviation of approximately 4.29. With a two-sided alpha of 0.05 and 80% power, approximately 18 participants per group were required. To account for uncertainty and potential data issues such as dropout, missing data, or low-quality fNIRS signals, a minimum target of 22 participants per group was set. Assuming 15% attrition, we planned to recruit 26 participants per group (total N = 52). Ultimately, 44 participants completed all assessments and were included in the main analysis (22 per group), meeting the minimum requirement for ANCOVA.

### 2.12 Statistical analysis

Statistical analyses were conducted on participants who completed the intervention and had valid outcome data (complete-case analysis). We performed statistical analyses using SPSS version 27.0 (IBM Corp., Armonk, NY, USA; Chinese version) and NirMaster. We assessed normality using the Shapiro–Wilk test. Approximately normally distributed data are presented as mean and standard deviation, whereas non-normally distributed data are presented as median and interquartile range. Categorical variables are reported as numbers and percentages. For baseline comparisons, we used independent-samples t-tests for continuous variables or Mann–Whitney U tests when assumptions were not met. For categorical variables, we used chi-square or Fisher’s exact tests.

For the primary outcome (BBS) and secondary outcomes (MBI, COP path length, and COP sway area), we used baseline-adjusted ANCOVA. Post-intervention values were treated as dependent variables, group as the fixed factor, and baseline values as covariates (Type III sums of squares). Results are reported as *F* values, *p* values, partial η^2^, adjusted between-group differences (Cloud Hands minus control), and 95% confidence intervals. We tested for homogeneity of regression slopes using the group-by-baseline interaction term. When this interaction was not significant, the model without the interaction was reported. For sensitivity analyses, we used change scores (*Δ* = post − baseline), with between-group comparisons conducted using the Mann–Whitney U test, and assessed within-group changes using the Wilcoxon signed-rank test.

For fNIRS connectivity analyses, we analysed Fisher r-to-z–transformed HbO connectivity values (Z). Baseline between-group comparisons across the 28 prespecified ROI-to-ROI connections were conducted using independent-samples t-tests, with Benjamini–Hochberg FDR correction applied to obtain q-values. Effect sizes (Cohen’s d) were calculated from post-intervention between-group comparisons to provide standardised estimates of group differences. For post-intervention analyses, we used baseline-adjusted ANCOVA with post-intervention *Z* values as the dependent variable, group as the fixed factor, and baseline *Z* values as the covariate. Results are reported as *F* values, *p* values, partial η^2^, adjusted between-group differences (experimental minus control), and 95% confidence intervals. To control for multiple comparisons across the 28 connections, FDR correction was applied to the ANCOVA results. A *q*-value below 0.05 was considered statistically significant. All tests were two sided, with *α* = 0.05. No imputation was performed for missing data because post-intervention outcome data were unavailable for participants excluded after randomisation. Analyses were therefore conducted using a complete-case approach, and no intention-to-treat analysis was performed.

## 3 Results

### 3.1 Participant flow and baseline characteristics

**Figure 2:**
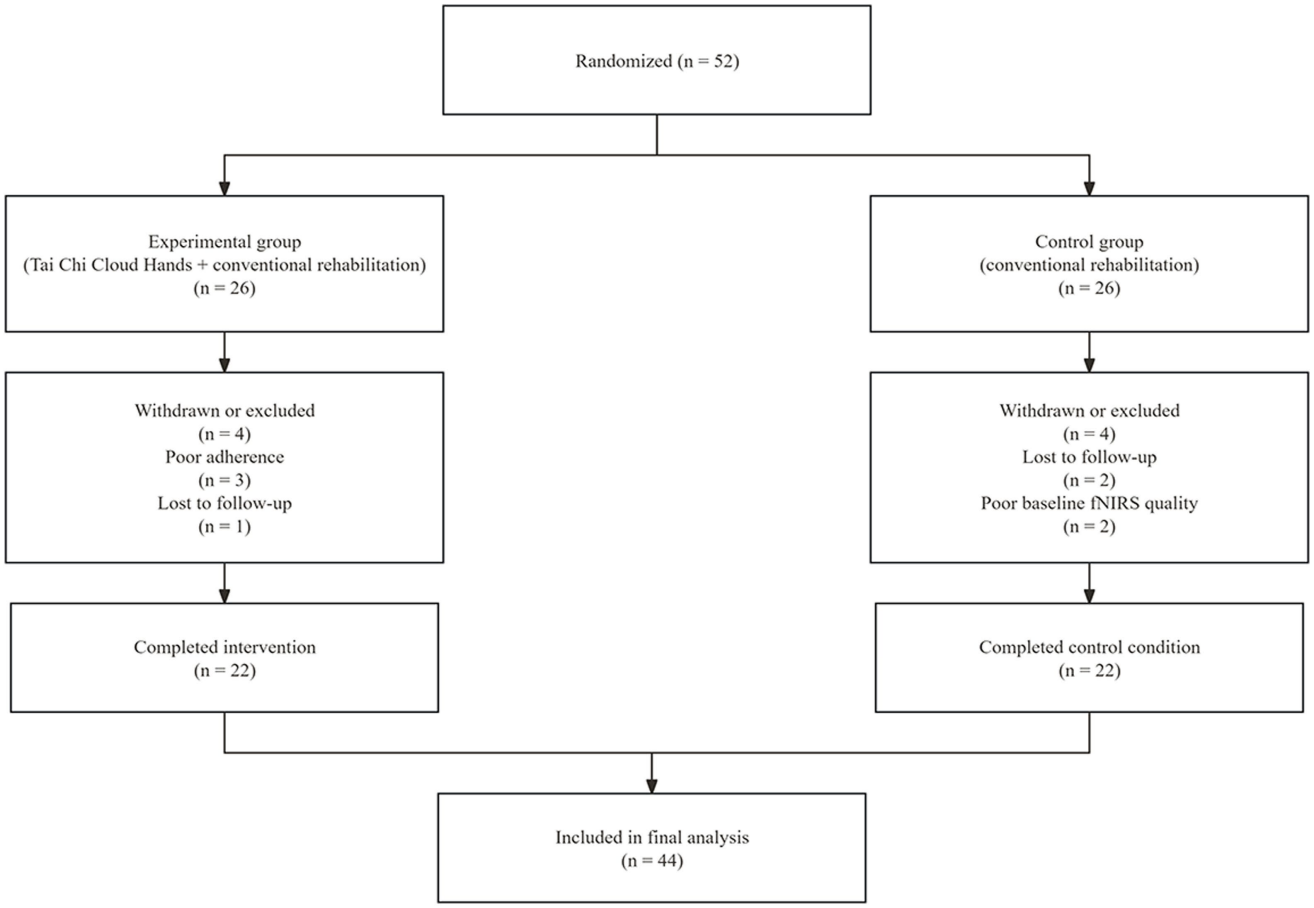
CONSORT flow diagram of participant enrolment, allocation, follow-up, and analysis. A total of 52 participants were randomised to the experimental group or the control group. During the intervention period, eight participants withdrew or were excluded. Reasons for withdrawal or exclusion are shown for each group. Finally, 44 participants completed the study and were included in the main analysis.

We enrolled 52 patients and randomised them to the experimental group (*n* = 26) or the control group (*n* = 26). During the intervention period, eight participants withdrew or were excluded from the study (four in each group). In the experimental group, three participants showed poor adherence to the intervention protocol, and one participant was lost to follow-up due to early discharge. In the control group, two participants were lost to follow-up due to early discharge, and two participants were excluded because of poor baseline fNIRS signal quality. The final analysis included 44 participants who completed all assessments (*n* = 22 per group). The participant flow throughout the trial is shown in Figure 2. Baseline demographic and clinical characteristics were similar between groups (all *p* > 0.05; see Table 1). No intervention-related or fNIRS-related adverse events were observed during the intervention period. FMA and LOS were not included in the formal study assessments because these outcomes had been removed from the formal study protocol after the pilot phase and before the start of the formal study. Therefore, no formal-study data on these measures were available for analysis.

### 3.2 Clinical scale outcomes (BBS and MBI)

After 2 weeks, both groups showed improvements in BBS and MBI scores compared to baseline scores (see Table 3). Baseline-adjusted ANCOVA supported the same conclusion, showing clear group effects for both outcomes (BBS: partial η^2^ = 0.420; MBI: partial η^2^ = 0.363). The adjusted between-group differences were 3.215 points for the BBS and 6.056 points for the MBI (see Table 3). Change-score comparisons showed greater improvement in the experimental group than in the control group (Δ*p* < 0.001; see Table 3). Sensitivity analyses using nonparametric tests yielded results consistent with the primary ANCOVA findings (see Supplementary Table 1). Additional responder analyses showed that all participants in the experimental group and 86.4% of participants in the control group achieved a clinically meaningful improvement of ≥4 points on the BBS. A larger improvement threshold (≥6 points) was observed in 90.9% of participants in the experimental group and 40.9% of participants in the control group (*χ*^2^ = 12.239, *p* < 0.001; see Supplementary Table 2).

**Table 3:** Clinical outcomes of balance and ADL (baseline-adjusted ANCOVA).

Outcome	Experimental (*n* = 22) baseline/post	Control (*n* = 22) baseline/post	Adjusted group difference (Exp−Ctrl), 95% CI	ANCOVA (*F*, *p*, ηp^2^)
BBS	35.86 ± 2.71	36.05 ± 3.42	3.215 (2.024, 4.407)	29.694, <0.001, 0.420
	44.68 ± 3.32	40.95 ± 3.57		
MBI	63.00 ± 4.65	66.05 ± 5.91	6.056 (3.527, 8.586)	23.379, <0.001, 0.363
	78.55 ± 5.06	74.05 ± 6.41		

Values are mean ± SD. *n* = 22 per group. ANCOVA df = (1,41); post-intervention score as dependent variable; baseline as covariate.

### 3.3 Static postural stability (COP path length and COP sway area)

COP path length and sway area decreased in both groups after the intervention (all *p* < 0.001; see Table 4). The experimental group showed a larger reduction in COP path length than the control group (*F* = 17.160, *p* < 0.001, partial η^2^ = 0.295; adjusted difference = −115.816 mm). The group difference was also significant for COP sway area (*F* = 5.172, *p* = 0.028, partial η^2^ = 0.112; adjusted difference = −79.480 mm^2^; see Table 4). Nonparametric sensitivity analyses yielded consistent results for postural stability outcomes (see Supplementary Table 1).

**Table 4:** Postural stability outcomes assessed by force platform (baseline-adjusted ANCOVA).

Outcome	Experimental (*n* = 22) Baseline/Post	Control (*n* = 22) Baseline/Post	Adjusted group difference (Exp−Ctrl), 95% CI	ANCOVA (*F*, *p*, ηp^2^)
COP path length (mm)	547.45 ± 143.997	541.91 ± 157.232	−115.816 (−172.279, −59.354)	17.160, <0.001, 0.295
	301.86 ± 125.055	414.50 ± 126.842		
COP sway area (mm^2^)	445.77 ± 170.045	491.82 ± 184.284	−79.480 (−150.060, −8.899)	5.172, 0.028, 0.112
	249.73 ± 113.593	351.91 ± 167.800		

Values are mean ± SD. *n* = 22 per group. ANCOVA df = (1, 41); post-intervention score as dependent variable; baseline as covariate.

### 3.4 Resting-state functional connectivity (ROI-to-ROI, HbO)

Baseline between-group comparisons across the 28 prespecified ROI-to-ROI connections are reported in Supplementary Table 3. Five connections showed significant differences after FDR correction (*q* ≤ 0.038). Therefore, baseline-adjusted ANCOVA was applied for all post-intervention comparisons.

**Figure 3:**
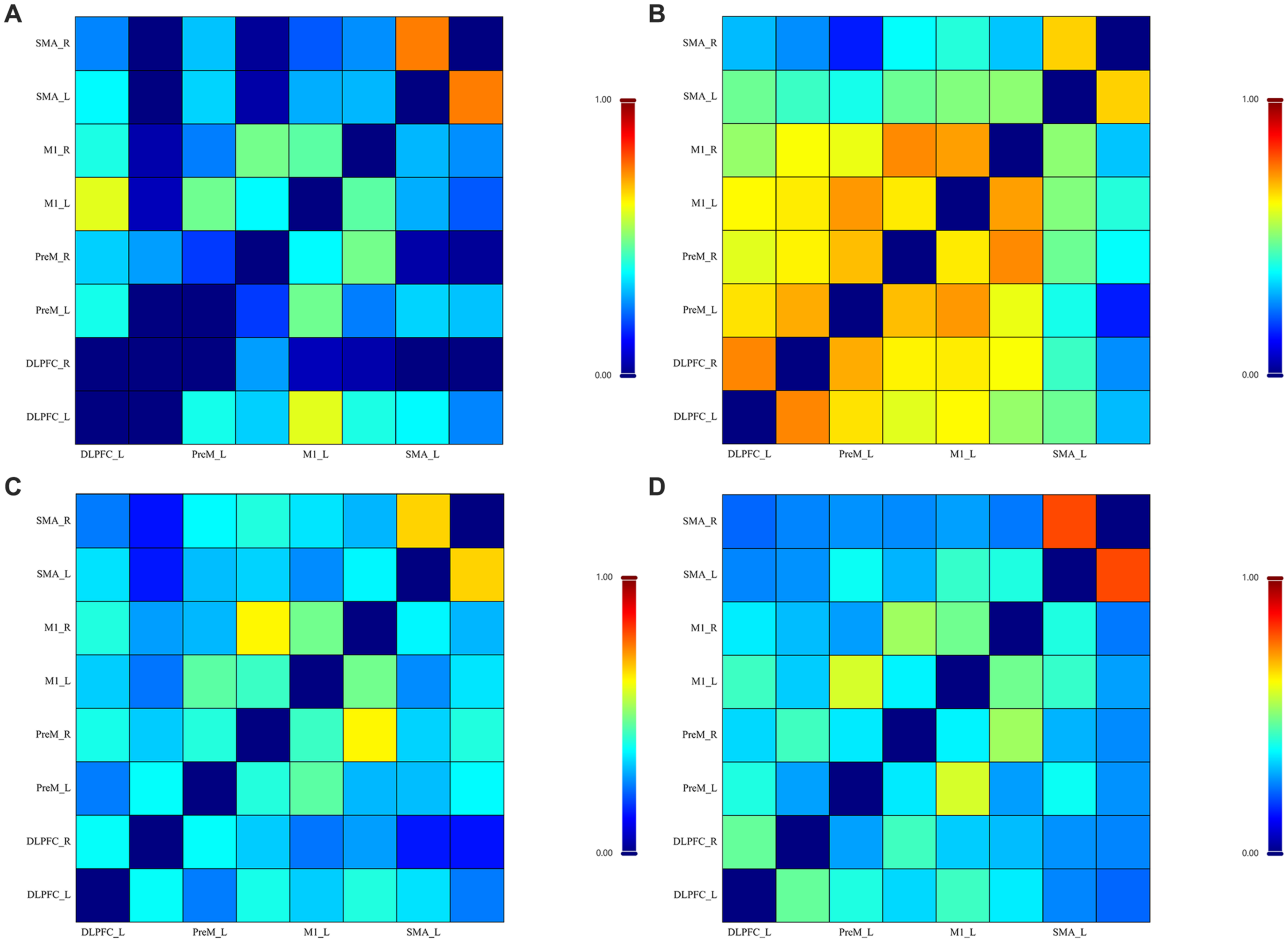
Resting-state fNIRS correlation (*r*) matrices. Resting-state functional connectivity matrices derived from fNIRS data after preprocessing in NirMaster. Colours indicate Pearson correlation coefficients (*r*), with warmer colours denoting stronger connectivity and cooler colours denoting weaker connectivity **(A)** Experimental group before intervention; **(B)** experimental group after intervention; **(C)** control group before intervention; and **(D)** control group after intervention.

ROI-to-ROI connectivity matrices showed an overall increase in connectivity after the intervention in the experimental group relative to baseline patterns (see Figure 3), and several connections remained significant after correction for multiple comparisons (see Table 5). In the control group, a similar pre–post pattern was observed (see Figure 3); however, no connections remained significant after FDR correction (*q* = 0.804–0.927; see Table 5).

**Table 5:** Resting-state functional connectivity (HbO) outcomes assessed by fNIRS (baseline-adjusted ANCOVA).

Connection	Experimental (*n* = 22) baseline/post	Control (*n* = 22) baseline/post	ANCOVA (*F*, *p*, ηp^2^)	FDR q	Adjusted group difference (95% CI)
L-DLPFC–R-DLPFC	0.036 ± 0.775	0.498 ± 0.619	7.805, 0.008, 0.160	0.017	0.411 (0.114, 0.709)
	1.017 ± 0.283	0.578 ± 0.585			
L-DLPFC–L-PreM	0.498 ± 0.410	0.294 ± 0.512	10.006, 0.003, 0.196	0.009	0.341 (0.123, 0.558)
	0.844 ± 0.322	0.480 ± 0.372			
L-DLPFC–R-PreM	0.407 ± 0.450	0.500 ± 0.470	11.118, 0.002, 0.213	0.009	0.325 (0.128, 0.521)
	0.706 ± 0.180	0.409 ± 0.455			
L-DLPFC–L-M1	0.738 ± 0.297	0.387 ± 0.376	5.15, 0.029, 0.112	0.048	0.348 (0.038, 0.657)
	0.854 ± 0.444	0.541 ± 0.446			
L-DLPFC–R-M1	0.458 ± 0.283	0.484 ± 0.317	2.563, 0.117, 0.059	0.172	0.182 (−0.048, 0.412)
	0.621 ± 0.300	0.441 ± 0.435			
L-DLPFC–L-SMA	0.421 ± 0.282	0.408 ± 0.458	6.123, 0.018, 0.130	0.033	0.252 (0.046, 0.457)
	0.554 ± 0.259	0.302 ± 0.395			
L-DLPFC–R-SMA	0.281 ± 0.303	0.302 ± 0.466	0.996, 0.324, 0.024	0.432	0.092 (−0.094, 0.278)
	0.339 ± 0.292	0.247 ± 0.311			
R-DLPFC–L-PreM	−0.125 ± 0.446	0.418 ± 0.368	27.968, 0.001, 0.406	0.009	0.742 (0.458, 1.025)
	0.909 ± 0.203	0.327 ± 0.526			
R-DLPFC–R-PreM	0.360 ± 0.548	0.381 ± 0.447	5.264, 0.027, 0.114	0.047	0.246 (0.029, 0.462)
	0.780 ± 0.222	0.537 ± 0.452			
R-DLPFC–L-M1	0.023 ± 0.699	0.275 ± 0.411	11.079, 0.002, 0.213	0.009	0.434 (0.171, 0.698)
	0.794 ± 0.228	0.359 ± 0.544			
R-DLPFC–R-M1	−0.003 ± 0.625	0.340 ± 0.439	10.184, 0.003, 0.199	0.009	0.406 (0.149, 0.664)
	0.770 ± 0.274	0.350 ± 0.491			
R-DLPFC–L-SMA	−0.143 ± 0.255	0.158 ± 0.413	0.232, 0.633, 0.006	0.656	0.066 (−0.211, 0.343)
	0.493 ± 0.294	0.328 ± 0.524			
R-DLPFC–R-SMA	−0.102 ± 0.236	0.166 ± 0.413	0.289, 0.594, 0.007	0.639	0.128 (−0.326, 0.189)
	0.292 ± 0.315	0.320 ± 0.453			
L-PreM–R-PreM	0.210 ± 0.394	0.472 ± 0.429	17.293, 0.001, 0.297	0.009	0.507 (0.261, 0.753)
	0.893 ± 0.269	0.443 ± 0.482			
L-PreM–L-M1	0.597 ± 0.401	0.630 ± 0.561	4.664, 0.037, 0.102	0.058	0.204 (0.013, 0.394)
	0.952 ± 0.207	0.752 ± 0.395			
L-PreM–R-M1	0.278 ± 0.338	0.364 ± 0.476	13.272, 0.001, 0.245	0.009	0.387 (0.172, 0.601)
	0.715 ± 0.144	0.346 ± 0.483			
L-PreM–L-SMA	0.372 ± 0.328	0.406 ± 0.534	0.000, 0.996, 0.000	0.996	0.000 (−0.176, 0.175)
	0.438 ± 0.257	0.438 ± 0.311			
L-PreM–R-SMA	0.345 ± 0.247	0.409 ± 0.314	2.203, 0.145, 0.051	0.203	−0.139 (−0.327, 0.050)
	0.177 ± 0.332	0.298 ± 0.295			
R-PreM–L-M1	0.427 ± 0.313	0.510 ± 0.409	20.603, 0.001, 0.334	0.009	0.397 (0.220, 0.573)
	0.784 ± 0.171	0.425 ± 0.429			
R-PreM–R-M1	0.623 ± 0.444	0.821 ± 0.360	10.578, 0.002, 0.205	0.009	0.340 (0.129, 0.550)
	0.963 ± 0.125	0.681 ± 0.482			
R-PreM–L-SMA	0.045 ± 0.257	0.397 ± 0.455	7.013, 0.011, 0.146	0.022	0.292 (0.069, 0.515)
	0.531 ± 0.133	0.381 ± 0.488			
R-PreM–R-SMA	0.025 ± 0.236	0.469 ± 0.344	10.112, 0.003, 0.198	0.009	0.395 (0.144, 0.646)
	0.443 ± 0.373	0.305 ± 0.357			
L-M1–R-M1	0.550 ± 0.330	0.605 ± 0.402	9.479, 0.004, 0.188	0.009	0.358 (0.123, 0.593)
	0.967 ± 0.299	0.625 ± 0.472			
L-M1–L-SMA	0.348 ± 0.375	0.318 ± 0.478	0.354, 0.555, 0.009	0.622	0.059 (−0.141, 0.259)
	0.592 ± 0.291	0.522 ± 0.421			
L-M1–R-SMA	0.245 ± 0.384	0.410 ± 0.378	1.742, 0.194, 0.041	0.259	0.131 (−0.070, 0.332)
	0.451 ± 0.195	0.335 ± 0.409			
R-M1–L-SMA	0.336 ± 0.271	0.431 ± 0.430	0.654, 0.423, 0.016	0.538	0.110 (−0.165, 0.385)
	0.617 ± 0.318	0.547 ± 0.580			
R-M1–R-SMA	0.314 ± 0.381	0.354 ± 0.424	0.382, 0.540, 0.009	0.622	0.084 (−0.190, 0.357)
	0.368 ± 0.457	0.298 ± 0.475			
L-SMA–R-SMA	1.061 ± 0.379	1.145 ± 0.838	11.912, 0.001, 0.225	0.009	−0.458 (−0.726, −0.190)
	0.906 ± 0.385	1.402 ± 0.634			

Values are Fisher r-to-z–transformed connectivity strength (*Z*). Between-group effects were estimated using baseline-adjusted ANCOVA (post-intervention *Z* as the dependent variable; group as a fixed factor; baseline *Z* as a covariate; df = 1, 41 for the group effect; only baseline *Z* was included as a covariate). *p* values for the group effect were corrected using FDR (Benjamini–Hochberg) across 28 prespecified connections.

After baseline-adjusted ANCOVA with FDR correction across the 28 prespecified connections (see Table 5), significant group differences mainly involved stronger DLPFC–PreM coupling (*q* ≤ 0.047), with the largest effect observed for R-DLPFC–L-PreM (*q* = 0.009); stronger interhemispheric PreM–M1 coupling (e.g., R-PreM–L-M1, *q* = 0.009); stronger interhemispheric M1–M1 coupling (L-M1–R-M1, *q* = 0.009); and weaker interhemispheric SMA–SMA coupling (L-SMA–R-SMA, *q* = 0.009). Some PreM–SMA connections also increased (*q* ≤ 0.022). Supplementary standardised effect sizes derived from post-intervention between-group comparisons showed heterogeneous magnitudes across connections, ranging from trivial to large (Cohen’s d). Some connections that showed relatively large post-intervention differences did not remain significant after baseline-adjusted ANCOVA.

Change-score sensitivity analyses for connections with baseline imbalance are presented in Supplementary Table 4. The direction and statistical significance of these effects were consistent with the primary ANCOVA results.

## 4 Discussion

### 4.1 Summary of findings

The results of the trial suggest that adding a 2-week, high-frequency Tai Chi Cloud Hands programme to conventional rehabilitation, under a higher total therapy dose, was associated with additional gains during inpatient care. Compared with conventional rehabilitation alone, the experimental group achieved greater improvements in balance and daily functioning and showed clearer changes in objective static postural stability. These findings are in line with the results of previous studies (24). Cloud Hands has also been evaluated in community-oriented programmes, with study protocols supporting its pragmatic delivery and standardisation (25). Recent randomised evidence further suggests that Cloud Hands variants, including assisted forms, can yield measurable motor benefits in people after stroke, supporting its rehabilitative plausibility as a structured training module (26). From a clinical feasibility perspective, Tai Chi-based stroke rehabilitation programmes have been reported to be implementable and potentially beneficial across symptom and functional domains, consistent with the inpatient add-on model used here (27).

### 4.2 Why the experimental group improved more

Both groups improved, which is expected with structured rehabilitation. The larger gains in the experimental group may reflect two related factors: greater overall practise and more task-relevant practise. The additional sessions increased repetition and overall dose, and motor-learning-aligned practise is considered an important driver of experience-dependent plasticity and functional recovery after stroke (28). Additionally, clearer specification and optimisation of intensity/dose/dosage are increasingly recognised as necessary for interpreting and scaling neurorehabilitation effects, and the Cloud Hands add-on directly increased active practise exposure (29). At the same time, Cloud Hands combines trunk rotation, continuous weight shifting, bilateral coordination, and breathing rhythm within a single movement sequence. Upper-limb movement is coordinated with centre-of-mass control; hence, the training demands map closely onto core components of postural control. This may partly explain why the added training was associated with better standing stability and higher scores on functional scales rather than only improving test performance, although this interpretation should be considered alongside the higher overall therapy dose received by the experimental group. Although the experimental group received a higher total therapy dose, the present study cannot distinguish Tai Chi–specific effects from increased training dosage and therapist attention. Additionally, responder analyses indicated that a larger proportion of participants in the experimental group achieved a ≥ 6-point improvement in BBS, suggesting a greater likelihood of clinically meaningful functional gains at the individual level. The adjusted between-group difference in BBS (3.215 points) was modest and did not exceed the lower bound of commonly reported minimal detectable change values. However, analysis of clinically meaningful improvement thresholds showed that a larger proportion of participants in the experimental group achieved improvements of ≥4 and ≥6 points. This pattern suggests that the clinical relevance of the intervention may be better reflected by the proportion of participants who achieve meaningful improvement rather than the mean between-group difference alone.

### 4.3 What COP adds beyond clinical scales

Clinical scales capture functional ability, but they remain observer-rated and may miss subtle changes in postural control. COP indices provide a complementary, instrument-based view, and established COP features (including path-based and area-based descriptors) are widely used to quantify quiet-stance balance output (30). In this study, reductions in COP path length and sway area indicate reduced postural sway during quiet stance, which matches the direction of improvement in the BBS and MBI. Together, these outcomes suggest that functional gains were accompanied by measurable changes in postural control output.

### 4.4 Interpreting the connectivity pattern

Balance impairment after a stroke often reflects disrupted coordination across multiple regions rather than a single focal deficit (31). Maintaining posture draws on executive/attentional control, motor planning, anticipatory postural adjustments, and motor execution (32). When automatic control is compromised, prefrontal contributions can increase during walking, consistent with compensatory recruitment patterns observed after stroke (33). More broadly, stroke walking studies synthesised in the fNIRS literature also emphasise prefrontal engagement, particularly under dual-task demands, which is consistent with the potential relevance of DLPFC-linked control for functional mobility (34). The greatest and most consistent changes in this study centred on DLPFC–PreM and PreM–M1 connections. This pattern is consistent with the idea that postural control and gait-related transitions involve preparatory and anticipatory adjustments, which are often affected after stroke (35). It is also worth noting that recovery does not necessarily imply that all connections increase. Interhemispheric motor-network coupling may relate to impairment severity in chronic stroke, which is consistent with the possibility that rehabilitation may involve redistributing and rebalancing coupling rather than uniform strengthening (36). In line with this framework, the present results also showed reduced interhemispheric SMA connectivity alongside strengthened prefrontal–motor coupling. Consistent with this perspective, rehabilitation has also been associated with changes in resting-state functional connectivity alongside functional improvement (37). In this study, the behavioural gains observed here may have been accompanied by changes in connectivity within the prefrontal–motor network. Supplementary standardised effect sizes showed that the magnitude of connectivity differences varied across connections, and some post-intervention differences were no longer significant after baseline adjustment. In addition, because resting-state fNIRS remains susceptible to residual systemic physiological influences, some observed between-group differences in connectivity may partly reflect non-neural vascular coupling rather than neural reorganisation alone. The present study relied on resting-state measurements without task-based confirmation or brain-behaviour modelling; therefore, these connectivity findings should be interpreted as associative rather than mechanistic.

### 4.5 Contribution relative to existing evidence

Earlier research shows that Tai Chi-based training can improve balance after a stroke and that structured practise can affect function. This study adds three key points. First, even a short and intensive 2-week protocol performed daily was associated with measurable improvements in clinical and instrument-based outcomes during the inpatient period. Second, results from clinical scales, COP measures, and resting-state fNIRS showed broadly convergent patterns, strengthening the overall findings whilst still requiring cautious interpretation of the connectivity results. Third, the connectivity results were adjusted for multiple comparisons, and the control group showed no major changes, which supports the consistency of the network-level findings within the present dataset.

### 4.6 Clinical implications

Cloud Hands does not require special equipment and can be easily standardised and repeated, making it a practical addition to regular balance training. In clinical practise, therapists should focus on safety by guarding against fatigue, monitoring cardiovascular tolerance, and gradually increasing the training dose. In line with earlier work on Tai Chi-based stroke rehabilitation highlighting practical considerations across functional domains, inpatient programmes should use clear instructions and gradual progression whilst prioritising safety.

### 4.7 Limitations and future directions

This study has some limitations. First, the sample size was small and derived from a single centre, and participants with both ischaemic and haemorrhagic strokes were included without stratification according to lesion location. Therefore, subtype-specific effects could not be examined. Second, the intervention lasted only 2 weeks, and no follow-up assessments were conducted, limiting conclusions about the durability of effects. Third, total training time and therapist contact differed between groups; therefore, some benefits may reflect dose or attention effects, and the present design does not allow the Tai Chi-specific contribution to be separated from the effects of greater overall therapy exposure. Fourth, analyses were based on complete cases only, and no imputation was performed for missing data. Because post-randomisation exclusions were related to adherence, early discharge, and baseline fNIRS signal quality, attrition bias cannot be completely excluded. Fifth, WHO primary registry registration was not completed before enrolment of the first participant because the study was redirected during the registration process to the International Traditional Medicine Clinical Trial Registry platform. In addition, FMA and LOS had been listed in an earlier registry version but were removed after the pilot phase and before the formal study began, and these outcomes were therefore not collected in the formal study. These issues should be considered when interpreting the protocol transparency of the study. Sixth, it was not possible to blind participants or therapists, and expectancy effects cannot be excluded. However, outcome assessments were conducted by blinded assessors to reduce measurement bias. Finally, fNIRS provides limited coverage of deep brain structures, and methods that aim to estimate deeper activity indicate that standard fNIRS predominantly reflects cortical signals (38). Additionally, resting-state fNIRS signals may be influenced by systemic physiological fluctuations such as scalp blood flow and vascular oscillations. Although preprocessing procedures were applied to attenuate these effects, the absence of short-channel regression and explicit physiological noise correction means that systemic physiological contributions cannot be completely excluded and may have influenced connectivity estimates. Accordingly, some observed between-group differences in resting-state connectivity may partly reflect residual systemic coupling rather than neural reorganisation alone. Moreover, resting-state fNIRS is influenced by systemic physiological factors, and removing these effects can alter resting-state network estimates and improve interpretability across individuals and time (39). Generally, fNIRS can yield false-positive or false-negative findings due to physiological and technical factors, underscoring the need for careful data processing and cautious interpretation (40).

Future research should include larger, multicentre samples and incorporate follow-up assessments. Time-matched attention controls or three-arm designs would help disentangle the effects of Cloud Hands from those of training dose. Including measures of dynamic balance, walking, and dual-task performance would enhance ecological relevance. In addition to examining specific regional connections, applying graph-theoretical metrics to resting-state fNIRS data may provide a more comprehensive view of network organisation. Given evidence that mind–body training can modulate resting-state functional connectivity in cognitive-control networks, a logical next step is to test whether similar connectivity targets emerge in post-stroke cohorts with balance deficits undergoing Cloud Hands training (41). Evidence linking intervention-related changes in resting-state connectivity to motor outcomes after stroke suggests that future studies should prioritise clear brain-behaviour models, such as prediction or mediation analyses, to strengthen mechanistic claims (42).

## 5 Conclusion

The addition of a 2-week intensive Tai Chi Cloud Hands programme to conventional rehabilitation, delivered at a higher total therapy dose, was associated with greater improvements in balance, daily functioning, and static postural stability in patients with post-stroke balance impairment. These improvements were observed alongside changes in resting-state connectivity within the prefrontal–motor network. However, because the present design did not control for total therapy dose, the observed additional benefits cannot be attributed specifically to Tai Chi independent of greater training dosage and therapist attention. In addition, because resting-state fNIRS connectivity remains susceptible to systemic physiological influences, these connectivity findings should be interpreted cautiously as associative rather than mechanistic. Given that the programme is straightforward to teach, standardise, and deliver without specialised equipment, it may serve as a practical adjunct to conventional balance rehabilitation.

## Data Availability

The raw data supporting the conclusions of this article will be made available by the authors, without undue reservation.
