# Supplementary material for: Effects of Tai Chi Cloud Hands on balance and resting-state functional connectivity after stroke: an fNIRS study
Source: Front Neurol. 2026 May 7;17:1791157. doi: 10.3389/fneur.2026.1791157 (PMC13189826; doi:10.3389/fneur.2026.1791157)
Supplement: Supplementary file 1 [file Table_1.DOCX]

Supplementary Material

| **Supplementary Table 1. Sensitivity analyses for clinical scales and postural stability outcomes (nonparametric tests)** | | | | | | |
| --- | --- | --- | --- | --- | --- | --- |
| **Outcome** | **Experimental**  **(within-group) Wilcoxon Z** | **P value** | **Control**  **(within-group) Wilcoxon Z** | **P value** | **Between-group**  **(Δ=Post−Baseline) Mann–Whitney Z** | **P value** |
| **BBS** | 4.115 | <0.001 | 4.128 | <0.001 | −4.369 | <0.001 |
| **MBI** | 4.112 | <0.001 | 4.113 | <0.001 | −4.393 | <0.001 |
| **COP path length (mm)** | −4.107 | <0.001 | -3.929 | <0.001 | −3.263 | 0.001 |
| **COP sway area (mm²)** | −3.912 | <0.001 | -4.015 | <0.001 | −2.371 | 0.018 |

Supplementary Table 1 Note：Values are Wilcoxon signed-rank (within-group, related samples) and Mann–Whitney U (between-group, independent samples) test statistics reported as standardized Z with two-sided P values. Δ indicates change score (Post−Baseline). n=22 per group.

| **Supplementary Table 2. Proportion of participants achieving clinically meaningful improvement in BBS.** | | | | | |
| --- | --- | --- | --- | --- | --- |
| **Threshold** |  | **Responder n (%)** | **Non-responder n (%)** | **χ²** | **P value** |
| **≥4 points improvement** | **Experimental (n=22)** | 22 (100.0%) | 0 (0.0%) |  |  |
|  | **Control (n=22)** | 19 (86.4%) | 3 (13.6%) | 3.220 | 0.073 |
| **≥6 points improvement** | **Experimental (n=22)** | 20 (90.9%) | 2 (9.1%) |  |  |
|  | **Control (n=22)** | 9 (40.9%) | 13 (59.1%) | 12.239 | <0.001 |

Supplementary Table 2 Note：Clinically meaningful improvement was defined as an increase of ≥4 points on the Berg Balance Scale (BBS), corresponding to the lower bound of the minimal detectable change. A higher improvement threshold of ≥6 points was also examined to represent larger functional improvement. Values are reported as number and percentage of participants achieving each threshold within each group. Group differences were tested using the chi-square test.

| **Supplementary Table 3. Baseline between-group comparisons of resting-state functional connectivity (HbO) between the experimental and control groups (ROI-to-ROI analysis).** | | | | | |
| --- | --- | --- | --- | --- | --- |
| **Connection** | **Experimental (n = 22)**  **Baseline mean ± SD** | **Control (n = 22)**  **Baseline mean ± SD** | **t (df = 42)** | **P value** | **FDR q** |
| **L-DLPFC–R-DLPFC** | 0.036 ± 0.775 | 0.498 ± 0.619 | -2.039 | 0.048 | 0.168 |
| **L-DLPFC–L-PreM** | 0.498 ± 0.410 | 0.294 ± 0.512 | 1.223 | 0.228 | 0.491 |
| **L-DLPFC–R-PreM** | 0.407 ± 0.450 | 0.500 ± 0.470 | -0.654 | 0.517 | 0.804 |
| **L-DLPFC–L-M1** | 0.738 ± 0.297 | 0.387 ± 0.376 | 3.427 | 0.001 | 0.013 |
| **L-DLPFC–R-M1** | 0.458 ± 0.283 | 0.484 ± 0.317 | -0.114 | 0.910 | 0.956 |
| **L-DLPFC–L-SMA** | 0.421 ± 0.282 | 0.408 ± 0.458 | -0.099 | 0.922 | 0.956 |
| **L-DLPFC–R-SMA** | 0.281 ± 0.303 | 0.302 ± 0.466 | -0.112 | 0.911 | 0.956 |
| **R-DLPFC–L-PreM** | -0.125 ± 0.446 | .418 ± 0.368 | -4.598 | < 0.001 | 0.001 |
| **R-DLPFC–R-PreM** | 0.360 ± 0.548 | 0.381 ± 0.447 | 0.040 | 0.969 | 0.969 |
| **R-DLPFC–L-M1** | 0.023 ± 0.699 | 0.275 ± 0.411 | -1.368 | 0.179 | 0.417 |
| **R-DLPFC–R-M1** | -0.003 ± 0.625 | 0.340 ± 0.439 | -1.839 | 0.073 | 0.228 |
| **R-DLPFC–L-SMA** | -0.143 ± 0.255 | 0.158 ± 0.413 | -2.889 | 0.007 | 0.038 |
| **R-DLPFC–R-SMA** | -0.102 ± 0.236 | 0.166 ± 0.413 | -2.455 | 0.018 | 0.086 |
| **L-PreM–R-PreM** | 0.210 ± 0.394 | 0.472 ± 0.429 | -2.191 | 0.034 | 0.137 |
| **L-PreM–L-M1** | 0.597 ± 0.401 | 0.630 ± 0.561 | -0.261 | 0.795 | 0.956 |
| **L-PreM–R-M1** | 0.278 ± 0.338 | 0.364 ± 0.476 | -0.754 | 0.455 | 0.749 |
| **L-PreM–L-SMA** | 0.372 ± 0.328 | 0.406 ± 0.534 | -0.272 | 0.787 | 0.956 |
| **L-PreM–R-SMA** | 0.345 ± 0.247 | 0.409 ± 0.314 | -0.935 | 0.355 | 0.663 |
| **R-PreM–L-M1** | 0.427 ± 0.313 | 0.510 ± 0.409 | -0.778 | 0.441 | 0.749 |
| **R-PreM–R-M1** | 0.623 ± 0.444 | 0.821 ± 0.360 | -1.541 | 0.131 | 0.367 |
| **R-PreM–L-SMA** | 0.045 ± 0.257 | 0.397 ± 0.455 | -3.097 | 0.004 | 0.029 |
| **R-PreM–R-SMA** | 0.025 ± 0.236 | 0.469 ± 0.344 | -4.894 | < 0.001 | 0.001 |
| **L-M1–R-M1** | 0.550 ± 0.330 | 0.605 ± 0.402 | -0.457 | 0.650 | 0.910 |
| **L-M1–L-SMA** | 0.348 ± 0.375 | 0.318 ± 0.478 | 0.152 | 0.880 | 0.956 |
| **L-M1–R-SMA** | 0.245 ± 0.384 | 0.410 ± 0.378 | -1.390 | 0.172 | 0.417 |
| **R-M1–L-SMA** | 0.336 ± 0.271 | 0.431 ± 0.430 | -1.003 | 0.322 | 0.643 |
| **R-M1–R-SMA** | 0.314 ± 0.381 | 0.354 ± 0.424 | -0.279 | 0.782 | 0.956 |
| **L-SMA–R-SMA** | 1.061 ± 0.379 | 1.145 ± 0.838 | -0.612 | 0.546 | 0.804 |

Supplementary Table 3 Note：Values are Fisher’s z-transformed resting-state connectivity coefficients at baseline (mean ± SD). Group differences were tested using two-tailed independent-samples t-tests (df = 42). False discovery rate (FDR) correction was applied across the 28 prespecified ROI-to-ROI connections. All tests were conducted at α = 0.05.

| **Supplementary Table 4. Change-score (ΔZ) sensitivity analyses for connections with baseline imbalance** | | | | | |
| --- | --- | --- | --- | --- | --- |
| **Connection** | **Experimental (n = 22)**  **ΔZ mean ± SD** | **Control (n = 22)**  **ΔZ mean ± SD** | **t (df = 42)** | **P value** | **Cohen’s d** |
| **L-DLPFC–L-M1** | 0.116 ± 0.469 | 0.154 ± 0.672 | -0.219 | 0.828 | -0.066 |
| **R-DLPFC–L-PreM** | 1.034 ± 0.393 | -0.091 ± 0.548 | 7.827 | < 0.001 | 2.360 |
| **R-PreM–L-SMA** | 0.486 ± 0.326 | -0.016 ± 0.449 | 4.239 | < 0.001 | 1.278 |
| **R-PreM–R-SMA** | 0.738 ± 0.297 | -0.164 ± 0.388 | 5.582 | < 0.001 | 1.683 |

Supplementary Table 4 Note：ΔZ = post-intervention (T1) minus baseline (T0) Fisher’s z-transformed connectivity. Group differences were tested using two-tailed independent-samples t-tests. Cohen’s d represents standardized mean differences. Sensitivity analyses were performed for connections that showed significant baseline differences after FDR correction. These analyses were conducted to verify that between-group effects were not driven by baseline imbalance.
