# Supplementary figures and images for: Effects of Tai Chi Cloud Hands on balance and resting-state functional connectivity after stroke: an fNIRS study
Source: Front Neurol. 2026 May 7;17:1791157. doi: 10.3389/fneur.2026.1791157 (PMC13189826; doi:10.3389/fneur.2026.1791157)

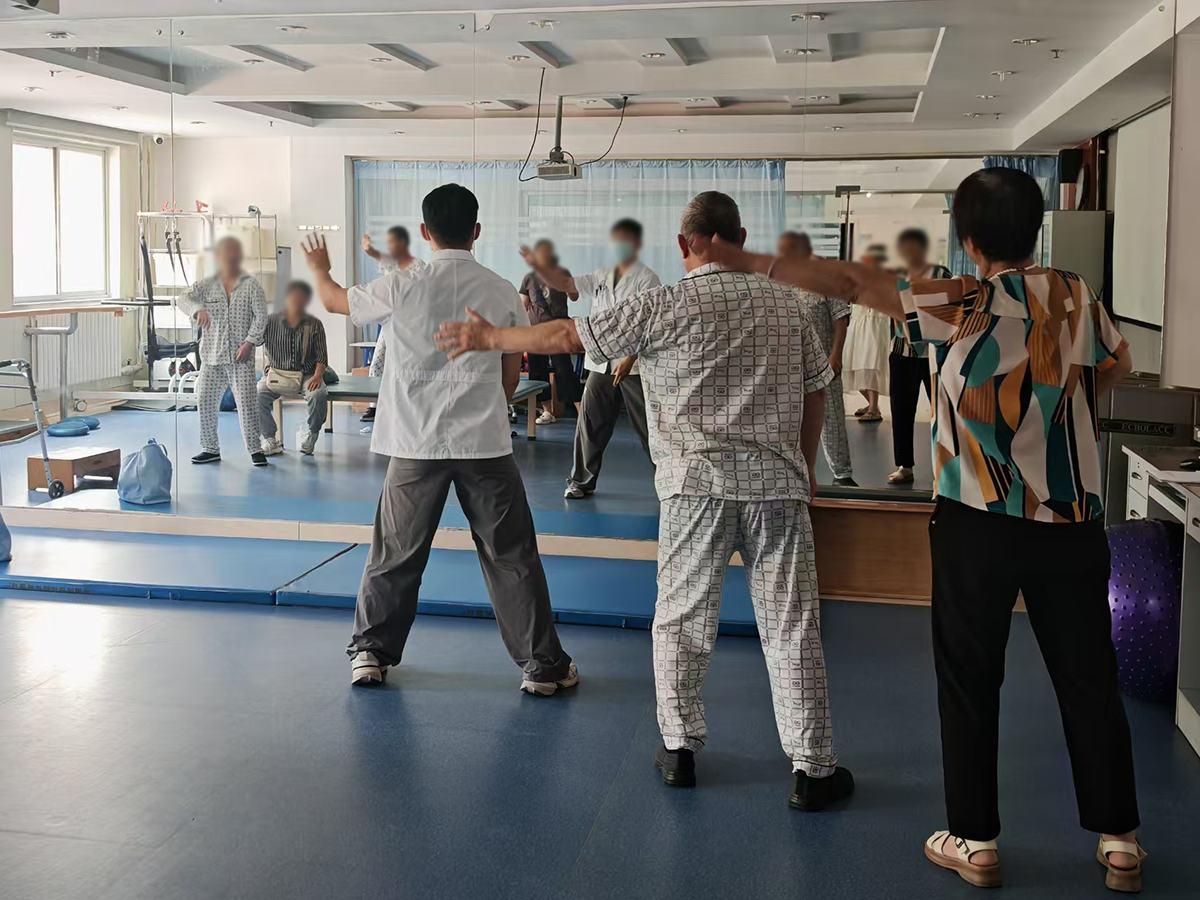

Supplement: Supplementary file 2 [file Image_1.TIF]
